# In Vitro Culture and Characterization of Human Lung Cancer Circulating Tumor Cells Isolated by Size Exclusion from an Orthotopic Nude-Mouse Model Expressing Fluorescent Protein

**DOI:** 10.1007/s10895-014-1439-3

**Published:** 2014-08-22

**Authors:** Katarina Kolostova, Yong Zhang, Robert M. Hoffman, Vladimir Bobek

**Affiliations:** 1 Department of Laboratory Genetics, University Hospital Kralovske Vinohrady, Srobarova 50, 100 34 Prague, Czech Republic; 2 AntiCancer, Inc., San Diego, CA USA; 3 Department of Surgery, University of California, San Diego, CA USA; 4 3rd Department of Surgery First Faculty of Medicine, Charles University in Prague and University Hospital Motol, Prague, Czech Republic; 5 Department of Histology and Embryology, Wroclaw Medical University, Wroclaw, Poland

**Keywords:** Lung cancer, Orthotopic, Circulating tumor cells, CTC, In vitro culture, CTC, MetaCell, Filtration, Size, Fluorescence, RFP

## Abstract

In the present study, we demonstrate an animal model and recently introduced size–based exclusion method for circulating tumor cells (CTCs) isolation. The methodology enables subsequent in vitro CTC-culture and characterization. Human lung cancer cell line H460, expressing red fluorescent protein (H460-RFP), was orthotopically implanted in nude mice. CTCs were isolated by a size-based filtration method and successfully cultured in vitro *on the separating membrane* (MetaCell®), analyzed by means of time-lapse imaging. The cultured CTCs were heterogeneous in size and morphology even though they originated from a single tumor. The outer CTC-membranes were blebbing in general. Abnormal mitosis resulting in three daughter cells was frequently observed. The expression of RFP ensured that the CTCs originated from lung tumor. These readily isolatable, identifiable and cultivable CTCs can be used to characterize individual patient cancers and for screening of more effective treatment.

## Introduction

The detection of circulating tumour cells (CTCs) in the peripheral blood of patients with solid epithelial tumors holds a great promise, and many exciting separation technologies have been developed over the past years [1].

However, detecting CTCs remain technically challenging. CTCs are present at very low concentrations of one tumor cell in the background of millions of blood cells. Extremely sensitive methods are required for their identification and characterization. Nevertheless, presence of CTCs could be an evidence for disease progress towards cancer dissemination [2, 3]. However, the role of CTCs as a disease marker may be unique under different clinical conditions and should be carefully interpreted. Clinical validation of these new biomarkers requires analysis of CTCs throughout the course of in vivo and in vitro studies.

Animal model studies using orthotopic metastatic models could help to identify the role of CTCs as potential biomarker, enabling mutational analysis and functional testing of metastasis expansion from CTCs.

In the present study, we demonstrate a method for CTC isolation based on size exclusion from an orthotopic nude mouse model of human lung cancer labeled with RFP (red fluorescent protein), using capillary-action-driven blood flow though porous membranes. The separating membranes can be used for in vitro culture of the CTCs immediately after CTCs- enrichment process.

## Materials and Methods

### Cell Culture

The human H460 lung cancer cell line, expressing RFP, used in this study has been described previously [4]. The cells were grown in RPMI-1640 medium supplemented with 10 % fetal bovine serum (FBS) and gentamicin (Life Technologies, Carlsbad, CA) to 70–80 % confluence as described previously [5].

### Orthotopic Model of H460-RFP in Nude Mice

**Fig. 1:**
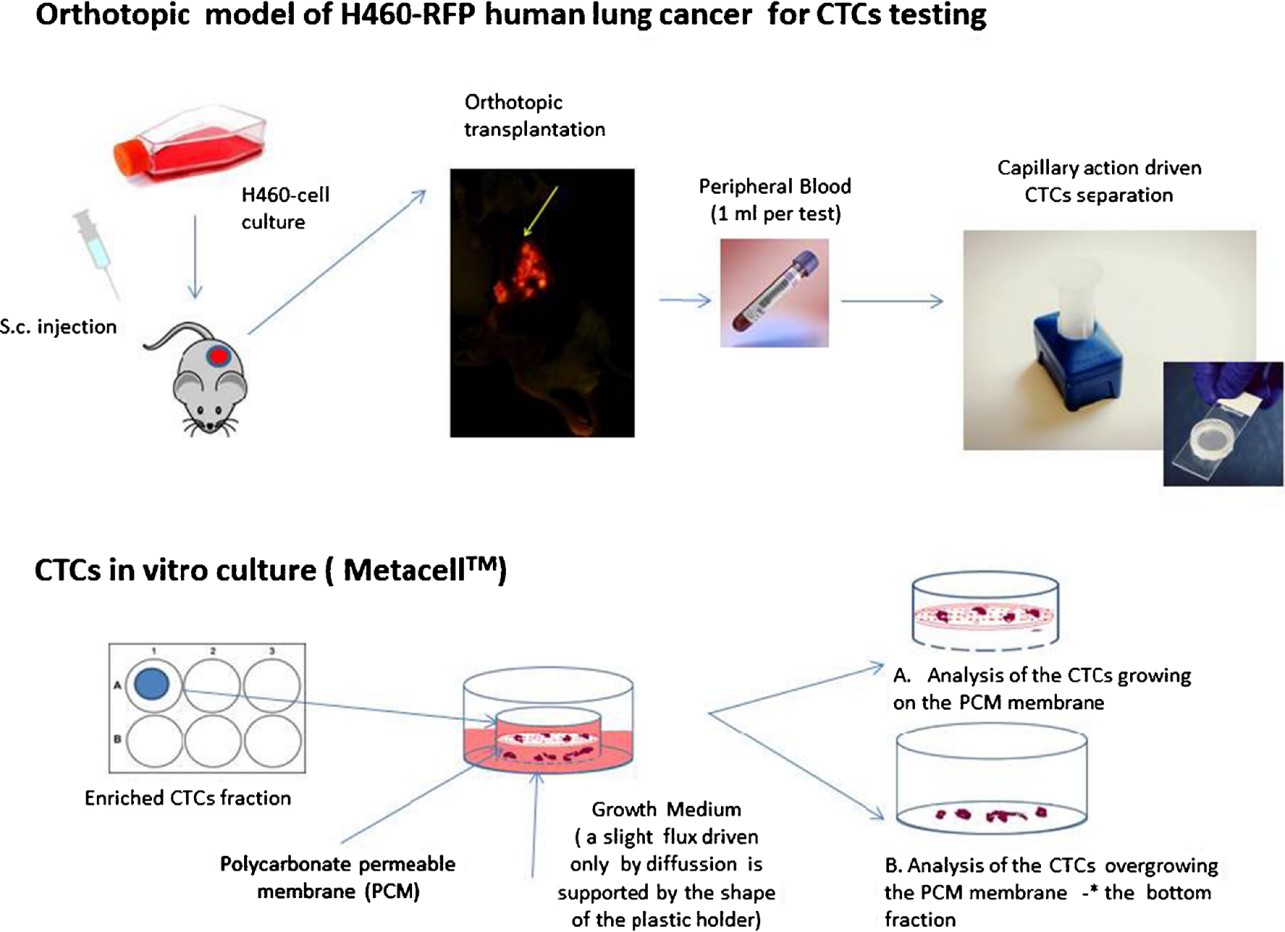
Experimental design

A subcutaneously-growing H460-RFP tumor fragment (1 mm^3^) from a single nude mouse was implanted by surgical orthotopic implantation (SOI) into the left lobe of the left lung in additional nude mice (group of eight animals). The animals were kept under isoflurane anesthesia during surgery. All procedures of the operation were performed with a 6.3× magnification microscope (Olympus, Tokyo, Japan), (Fig. 1).

Orthotopically-growing H460-RFP demonstrates aggressive metastatic behaviour [4]. Animal experiments were carried out in accordance with the Guidelines for the Care and Use of Laboratory Animals under NIH Assurance No. A3873-1.

### Isolation and Culture of the Circulating Tumor Cells from Blood

Blood (0.5–1.0 ml) was obtained from the nude mouse by cardiac puncture 1 month after orthotopic implantation of H460-RFP. The blood was placed in an EDTA tube (BD). A size-based separation method for viable CTC- enrichment (MetaCell®, Ostrava, Czech Republic) by filtration of the peripheral blood (PB) through a porous polycarbonate membrane (8 μm diameter pores) was used.

The membrane filters along with the plastic ring were transferred to 6-well tissue culture dishes (Fig. 1). RPMI 1640 medium (4 ml) with 10 % FBS is added on top of the filter, and CTCs were then cultured on the membrane at 37 °C, 5 % CO_2_. After 14 days, the CTCs were cultured directly on the plastic dish surface or microscopic slides (Lab-Tek Chambered Coverglass, Thermo Fisher Scientific, Rochester, U.S.) for further confocal microscopy analysis.

### Fluorescence Imaging

The Olympus OV100 Small Animal Imaging System (Olympus Corp., Tokyo, Japan) was used for bright-field and fluorescence imaging of the mice with orthotopically-implanted H460-GFP. The OV100 has a sensitive CCD camera and four objective lenses, parcentered and parfocal, enabling imaging from macrocellular to subcellular [6].

### Confocal Imaging

A Leica (Wetzler, Germany) TCS SP5 AOBS confocal microscope was used along with a DFC350 FX Digital Camera for 72 h time-lapse imaging.

## Results

### CTC Isolation

In the present report, we used a capillary–action-driven size-based separation of the CTCs from the peripheral blood of orthotopic models of human tumors using the MetaCell^®^ device. A schematic work- flow is shown on the Fig. 1. The CTCs were detected and separated in PB of all animals.

**Fig. 2:**
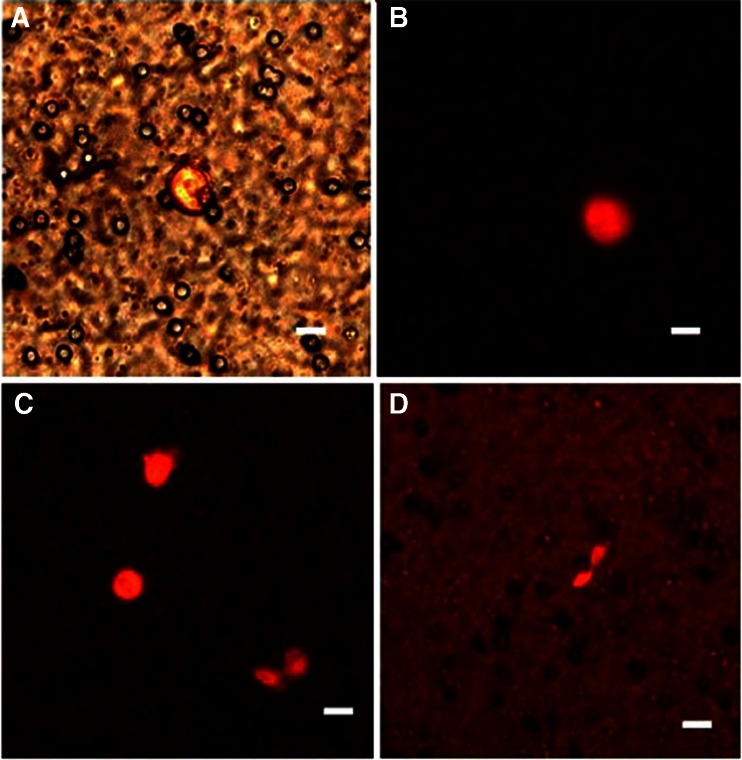
CTCs enriched from the peripheral blood (PB). PB was withdrawn from a mouse bearing H460-RFP human lung cancer growing orthotopically, 1 month after implantation. CTCs were detected on the microporous polycarbonate filter membrane by RFP-fluorescence. **a**, **b** 6 h after blood withdrawal. **c**, **d** 24 h after blood withdrawal. The first mitoses are observed already after 24 h. The CTCs were kept in RPMI medium with FBS. Scale bar 10 μm (Leica confocal microscope TCS SP5 AOBS)

Due to the gentle blood flow within the separation process, caused by natural forces of capillary action, the enriched CTCs remained viable on the separating polycarbonate membrane (Fig. 2). The viability is evidenced by the expression of RFP-protein in the captured cells (Fig. 2b, c, d).

**Fig. 3:**
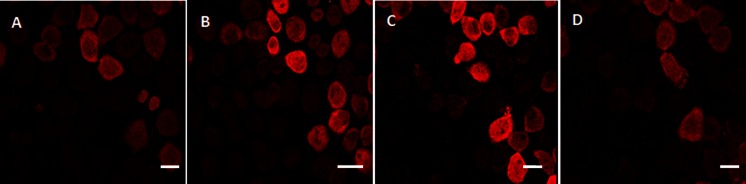
CTCs isolated from a tumor bearing mouse (orthotopically-transplanted human H460-RFP), cultured in vitro. The subsequent growth of confluent CTC-culture is shown derived from time -lapse imaging within 72 h. Scale bar 20 μm

The CTCs enriched on the membrane, which is hold in a plastic ring, were then placed directly into a 6-well culture dish along with cell culture medium (Fig. 1). The cells were scanned every 12 h and the first mitoses have been observed after 24 h (Fig. 2c, d). Using this method, we obtained confluent H460-RFP CTCs culture within several days. The subsequent growth of confluent CTC-culture is shown on Fig. 3. Figure 3 is derived from time–lapse imaging.

### CTC-Cytomorphology and Characteristics

**Fig. 4:**
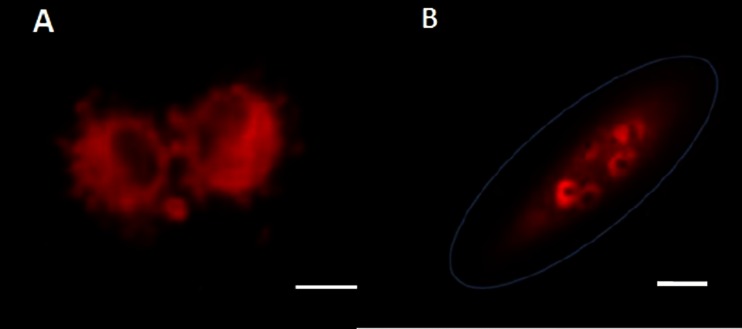
CTC -proliferation evidenced in vitro, in the culture of CTCs, isolated from an H460 experimental human lung cancer mice model. **a** Cell membrane vesicles starting to be produced within 3 days of culture. **b** Multi-nuclear stage can be also found very often in the CTCs. The CTCs were kept in RPMI medium with FBS. Scale bar 10 μm (Leica confocal microscope TCS SP5 AOBS)

Generally, the cultured CTCs exhibited blebbing surfaces (Fig. 4a), the cell membrane vesicles (blebs) are exhibited already within 3 days of culture. Another typical characteristics of in vitro growing CTCs is multinuclear stages with prominent nucleoli (Fig. 4b).

**Fig. 5:**
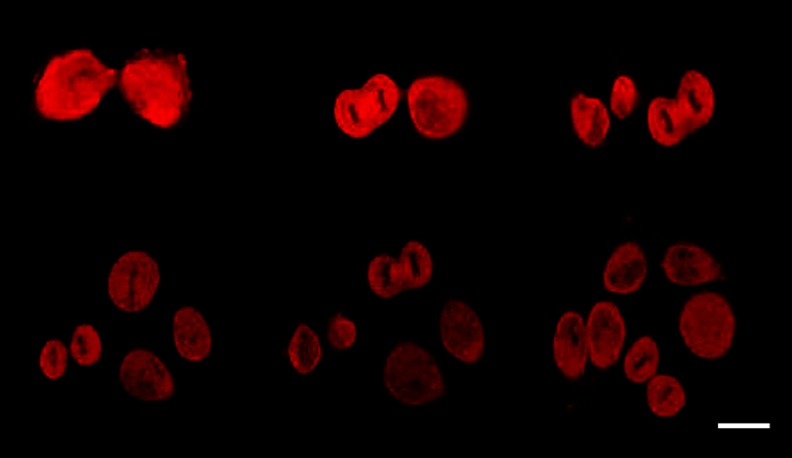
CTC proliferation monitored in vitro, in the culture of CTCs isolated from an H460 experimental human lung cancer mice model-confocal time-lapse imaging. In vitro culture was started from a single CTC-cell. One mitotic cycle took approximately 12 h in average. Scale bar (Leica confocal microscope TCS SP5 AOBS)

The CTCs appeared very heterogeneous even if originating from one primary tumor. They differed in size (11–30 μm) and proliferation capacity as evidenced by the time-lapse imaging (Fig. 5). Based on the time lapse imaging of the cells in vitro we may hypothesize that the cells use the blebs on the cell surface for faster movement if compared to the cells without blebbing. Morphologically, there is a difference between the CTC-culture and the original H460–RFP cell culture in the amount of produced blebs on the cell surface. The CTCs produce usually more blebs.

**Fig. 6:**
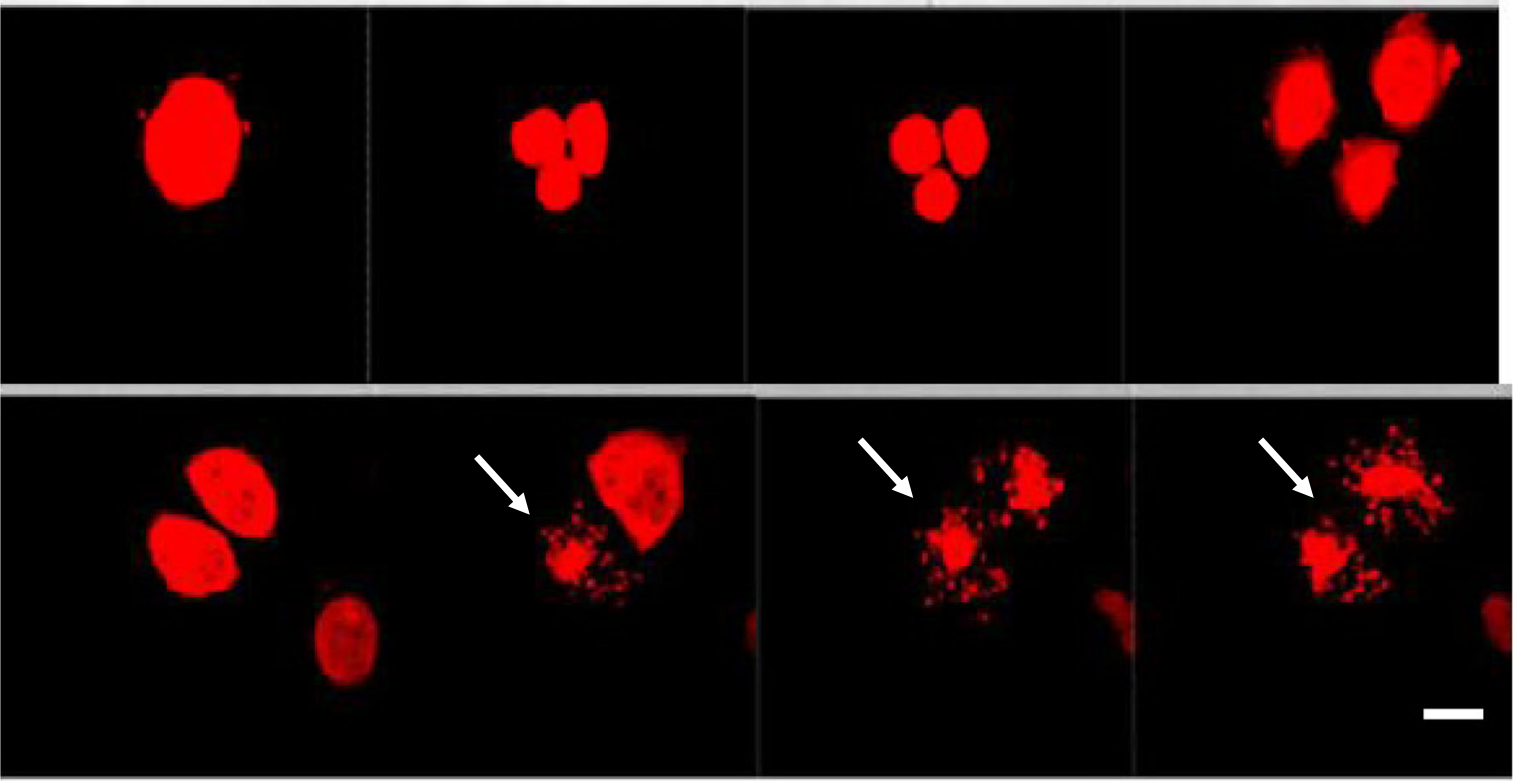
CTCs isolated from a tumor bearing mouse (orthotopically-transplanted human H460-RFP), cultured in vitro, imaged by time lapse imaging for 72 h. An irregular mitosis of one dividing cell (in the multinuclear stage) is shown on the picture. Subsequently three daughter cells developed. Each daughter-cell divided within the next mitotic cycle into 3 cells. Afterwards an apoptosis process started (see *arrows*). The CTCs were kept in RPMI medium with FBS. Scale bar 10 μm (Leica confocal microscope TCS SP5 AOBS)

Following, irregular CTC-divisions were seen very often; when one CTC-cell divided into the 3 daughter cells (Fig. 6). The cell originating from these unbalanced divisions may harbor a potential to divide again into three daughter cells within the next mitotic cycle. As shown on the Fig. 6, some of the 3 daughter cells may enter apoptotic process immediately after division.

The uncontrolled growth of the CTCs population, with many irregular mitoses, is in fact generating a population of the cancer cells significantly different to the primary cell culture. The CTCs in vitro cultures are definitely worth to be studied subsequently by immunophenotypization, gene expression profiling and mutational analysis.

## Discussion

Our aim was to develop a successful CTC isolation method and CTCs in vitro culture protocol enabling cytomorphological analysis of viable CTCs. Cytomorphological characterization of CTCs can be followed by further downstream analysis (e.g., karyotype analysis, gene expression analysis of chemoresistance genes, in vitro chemoresistance monitoring). All the information on CTCs characteristics can be used for personalizing cancer treatment [7].

Previously, we have shown the feasibility of culturing CTCs directly from whole blood using orthotopic models of human tumors expressing GFP (green fluorescent protein) using immunomagnetic separation of the CTC cells [5].

In the present study, CTCs were isolated from the H460- RFP orthotopic metastatic nude-mouse model of lung cancer by a size-based separation method. The majority of CTC isolation methods uses epithelial cell-adhesion molecule (EpCAM) to capture CTCs and cytokeratin (CK) antibodies to identify CTCs [8]. The weakness of these approaches could be that the rare cells isolated via EpCAM and/or cytokeratin (CK) binding may be circulating epithelial cells and more aggressive cell subpopulations might not have been isolated at all because they are EpCAM and CK negative [9, 10]. There is a broad morphological and immunophenotypical variation within CTCs derived from the same tumor type as we have realized during our observation.

The EpCAM-dependance could be one of the reasons why significant differences in separation efficiency have been reported if the immunomagnetic isolation of lung cancer CTCs (CellSearch®) was compared to size-based filtration CTC-capture (ISET®) [11].

Therefore, accurate detection of CTCs based on morphological features such as size could be crucial. Since only a limited number of CTCs are captured by all, it is important to expand the isolated CTCs in vitro in order to perform subsequent functional analyses.

CTCs have been found to create doublets and clusters in the blood [12, 13]. Circulating tumor microemboli have been observed in the peripheral blood of metastatic lung cancer, as well [11, 14]. The presence of clusters of CTC might be a relevant prognostic factor for malignancy [15]. However, large- and variable-sized clusters may not be isolated by immunomagnetic methods, since clusters may lack sufficient expression of EpCAM and/or CK. CTCs within clusters may also be surrounded by blood cells and thus, cannot be detected or recognized by immunogenic techniques [14, 16]. An advantage of size-based separation methods could be enabling the cluster isolation as well.

But there are also differences in the capture efficiency for the recently introduced size- based separation methods (e.g. ISET®, ScreeCell®, CellSieve®, MetaCell®). So far no comparison study has been conducted in the field of size-based separation methods. The most important difference between the reported size-based separation methods is that not all of them enable to capture viable CTCs.

On the other hand methodologies reporting viable CTC-cells enrichment have also not shown until today, how many out of the captured CTCs do survive the isolation process and are able to proceed towards mitotic process. The analytical reports evidencing the CTC- viability are generally based on the isolation of the cancer cells out of cell cultures diluted in the blood samples. One could expect, that these cells will behave differently, than CTCs isolated from patients’ blood or animal model.

We believe that data presented in this article, based on the use of orhotopic metastatic models, could be an unique in vivo proof of the viable CTCs separation principle.

One could hypothesize, that the use of orthotopical metastasis models could enhance the validation process for the size–based separation methods, in general. Having more viable CTCs leads us faster towards personalized cancer treatment.

We hope that combination of SOI technique with a use of cancer cell lines expressing fluorescent protein and subsequent detection of CTCs/clusters by size-based separation method (MetaCell®) could provide easier way for new anticancer agents testing. The count of CTCs appears to monitor the response to the treatment, in as little as a few weeks [13, 16]. Beyond an in vitro number count, an ex vivo functional study on patient-derived CTCs might provide a support for an immediate treatment decision regarding drug resistance [11].

In vitro culturing of CTCs is a base for the proliferation tests comparing the chemosensitivity. This study demonstrate a successful culturing of CTCs isolated from animal using filtration device (MetaCell®), which is as gentle as enabling obtaining CTCs with very high viability. These protocols can focus on using the CTCs culture for personalizing the oncological treatment in future.

## Conclusion

In vitro culturing of CTCs is a base for the proliferation test reporting the chemosensitivity. This study demonstrate a successful culturing of animal CTCs by use of filtration device (MetaCell®), which is as gentle as enabling obtaining CTCs with very high viability. These protocols can focus on using the CTCs culture for personalizing the oncological treatment in future.
